# Evaluation of the Effect of Optic Nerve Compression by Craniopharyngioma on Retinal Nerve Fiber Layer Thickness in Pediatric Patients

**DOI:** 10.3390/cancers17152574

**Published:** 2025-08-05

**Authors:** Klaudia Rakusiewicz-Krasnodębska, Agnieszka Bogusz-Wójcik, Elżbieta Moszczyńska, Maciej Jaworski, Paweł Kowalczyk, Wojciech Hautz

**Affiliations:** 1 Department of Pediatric Ophthalmology, Children’s Memorial Health Institute, 04-730 Warsaw, Poland; k.rakusiewicz@ipczd.pl (K.R.-K.); w.hautz@ipczd.pl (W.H.); 2 Department of Pediatric Endocrinology and Diabetology, Children’s Memorial Health Institute, 04-730 Warsaw, Poland; a.moszczynska@ipczd.pl; 3 Department of Clinical Biochemistry, Children’s Memorial Health Institute, 04-730 Warsaw, Poland; m.jaworski@ipczd.pl; 4 Department of Pediatric Neurosurgery, Children’s Memorial Health Institute, 04-730 Warsaw, Poland; p.kowalczyk@ipczd.pl

**Keywords:** retinal nerve fiber layer, optic chiasma, compressive optic neuropathy, optic coherence tomography, craniopharyngioma

## Abstract

Purpose: The present study aims to evaluate alterations in the peripapillary retinal nerve fiber layer (RNFL) thickness in pediatric patients following surgical resection of childhood-onset craniopharyngioma (CP) and to identify tumor characteristics and other factors influencing these alterations, including changes in the lesion’s location. Design: retrospective clinical cohort study. Methods: A retrospective analysis was conducted on 73 eyes from 38 patients with CP and 64 eyes from 32 age- and sex-matched healthy controls. The mean age of the CP patients was 10.3 ± 4.2 years (range 4–17), while the control group had a mean age of 10.5 ± 3.1 years (range 4–17). Optical coherence tomography (OCT) was used to assess the peripapillary RNFL thickness in the study and control groups. RNFL thickness was analyzed in the superior, inferior, and average sectors, as well as across eight optic nerve sectors. Tumor characteristics were evaluated to determine their correlation with changes in RNFL thickness in individual sectors. Results: Postoperative thickness of peripapillary RNFL in all individual sectors was significantly reduced in the CP group compared to healthy controls. Location, tumor volume, maximum tumor diameter, calcification, ventriculoperitoneal shunt, surgery technique, total resection, presence of Rosenthal fibers, and reoperation due to progression or recurrence correlated with damage to RNFL. Conclusions: CP is associated with significant reductions in RNFL thickness, indicating the tumor’s impact on optic nerve fibers. OCT is a valuable tool for monitoring visual pathway impairment and postoperative outcomes. Correlations between RNFL thickness in individual sectors and clinical parameters may offer valuable insights for diagnosis and monitoring, underlining their potential role in predicting visual outcomes. Regular RNFL evaluation should be integrated into the long-term care of CP patients to optimize visual prognosis and detect progressive or residual damage.

## Simple Summary

Childhood-onset craniopharyngioma is a rare brain tumor that can press on the optic chiasm and optic nerves responsible for vision, often leading to serious visual problems. In this study, we used a non-invasive imaging technique called optical coherence tomography to measure the thickness of the nerve fibers around the optic disc in children who had undergone surgery to remove this type of tumor. We compared our results with those of a group of healthy children of the same age. We found that children with a history of craniopharyngioma had significantly thinner nerve fibers in all areas of the optic nerve. The degree of nerve damage was related to the size, location, characteristics of the tumor, as well as the surgical method used, and the need for further treatment. Our findings show that the tumor and its treatment can cause lasting damage to the visual pathways. Monitoring the thickness of these nerve fibers can help doctors better understand how the tumor has affected a child’s vision and may help guide treatment and follow-up care. This simple and safe test could play an important role in protecting and preserving vision in children affected by this condition.

## 1. Introduction

Childhood-onset craniopharyngioma (CP) is a rare, benign yet locally aggressive tumor that originates in the sellar and parasellar regions of the brain. These tumors are closely associated with significant morbidity due to their anatomical location near critical structures, including the optic chiasm, hypothalamus, and pituitary gland [1,2,3,4,5,6]. Visual impairment is one of the most common and debilitating symptoms of CP [5,6,7,8]. Approximately 62–84% of patients with CP present with initial symptoms related to vision problems, such as decreased visual acuity, visual field defects (bitemporal hemianopia), or optic disc edema, often leading to the diagnosis [7,8,9,10]. Furthermore, visual impairment has a significant impact on patients’ daily functioning and quality of life after CP treatment [9]. Visual status at the diagnosis is a key determinant of long-term visual outcomes during follow-up after tumor regression [9,10]. The prevalence of persistent visual disturbances following surgery in pediatric CP patients ranges from 48% to 75% [2]. The most significant cause of vision problems is the suprasellar enlargement of the tumor, which exerts mechanical pressure on the optic chiasm and optic nerves. Prolonged compression can result in irreversible damage to the optic nerve cells, including retinal ganglion cells and their axons, leading to thinning of the retinal nerve fiber layer (RNFL). This compression may cause various visual disturbances, such as reduced visual acuity, visual field defects, and changes to the optic disc, including swelling or atrophy [5,7,11,12,13,14,15]. Early identification and monitoring of these changes are critical, as the degree of optic nerve damage often correlates with the severity of visual loss and the potential for postoperative recovery [15,16,17,18]. Advances in imaging technologies, particularly optical coherence tomography (OCT), have provided clinicians with a powerful, non-invasive tool to assess optic nerve health. OCT enables precise measurement of RNFL thickness, allowing for the detection of subtle changes in optic nerve integrity that may not be apparent on clinical examination alone, as well as the assessment of the optic nerve disc in the fundus. Especially in children, performing a standard visual field test can be challenging due to limited cooperation and attention; therefore, OCT is an objective and feasible diagnostic tool in the pediatric population. In cases of CP, OCT has emerged as a valuable method for assessing the extent of optic nerve damage, both pre- and postoperatively, during long-term monitoring and follow-up [19,20,21,22,23]. Despite its utility, the relationship between optic nerve compression and RNFL thinning in pediatric CP patients is not fully understood. Factors such as tumor size, morphology, and degree of chiasmal compression, as well as the timing and type of surgical intervention, may all influence the degree of optic nerve damage. Additionally, postoperative outcomes can be affected by endocrine dysfunction and other systemic complications commonly associated with CP [7,11,24,25,26]. This study aimed to investigate the influence of clinical parameters and tumor features associated with optic chiasm compression on the RNFL thickness in pediatric patients undergoing surgery for CP. Until now, data on tumor characteristics and their impact on the degree of RNFL damage in such a large group of pediatric patients with CP have not been analyzed. Gaining a deeper understanding of the mechanisms underlying optic nerve damage and identifying the relationships between contributing factors is essential for optimizing the treatment of CP, enhancing monitoring accuracy, and ultimately improving the quality of life for affected children.

## 2. Methods

This single-center, observational, retrospective, cross-sectional study included patients with early-onset CP treated at the Children’s Memorial Health Institute in Warsaw, Poland, between June 2021 and September 2024. The primary inclusion criteria were age below 18 years and the presence of a sellar tumor on pituitary magnetic resonance imaging (MRI), with or without chiasmal compression, treated surgically, with histopathologically confirmed adamantinomatous CP. The study protocol was approved by the Institutional Bioethics Committee of the Children’s Memorial Health Institute in Warsaw (21/KBE/2024) and was conducted by the principles of the Declaration of Helsinki. Written informed consent was obtained from all patients aged 13 years or older, as well as from the legal guardians of those under 13, after thoroughly explaining the study protocol, its nature, and potential risks.

The study group consisted of 73 eyes from 38 children, with a mean age at the time of OCT examination 10.3 ± 4.2; range 4–17, both 22 male and 16 female, who had undergone neurosurgical treatment for CP and were treated in the Endocrinology Department, with ophthalmological evaluations conducted in the Ophthalmology Department of the Children’s Memorial Health Institute. The inclusion criterion for the study group was histopathological confirmation of adamantinomatous CP in the postoperative tissue. The control group included 64 eyes from 32 healthy children with no diagnosis of CP or other systemic diseases. The eyes were matched for gender and age (mean age at the OCT examination: 10.5 ± 3.1 years; range: 4–17 years), comprising 12 male and 20 female participants.

Patients with ocular diseases, those who had undergone ophthalmic surgery, or those with concomitant ophthalmic conditions such as glaucoma, hereditary retinal dystrophies, optic nerve diseases, retinal diseases, significant corneal or lens opacities, any macular disease, or a previous diagnosis of glaucoma were excluded. Additionally, patients with significant refractive errors greater than 3 diopters (D) were also excluded. Exclusion criteria for both groups included previously diagnosed and treated systemic diseases, such as diabetes mellitus, isolated hypertension, renal disease, neurological disorders, and a history of prematurity, as well as all conditions with proven effects on RNFL. All patients in both the study and control groups underwent a comprehensive ophthalmological examination, which included assessment of best-corrected visual acuity (BCVA), slit-lamp biomicroscopy of the anterior segment, and fundus examination following mydriasis with 1% Tropicamide.

**Figure 1:**
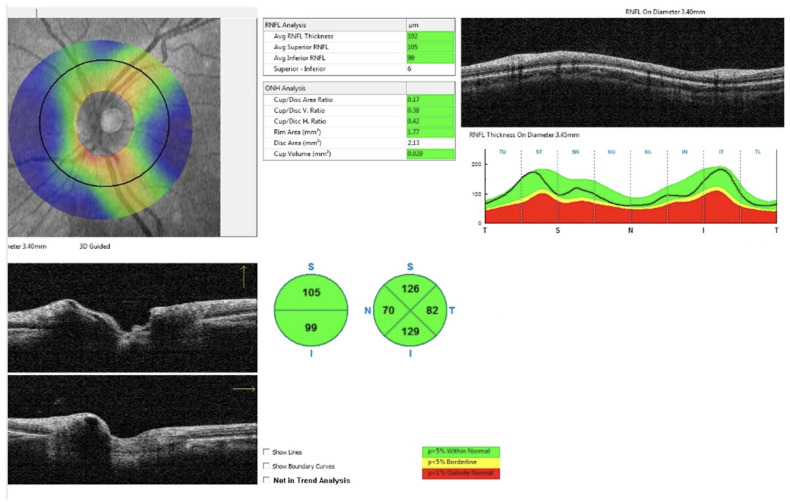
Peripapillary RNFL thickness analysis using spectral-domain OCT. The RNFL thickness map (**top left**), circular B-scan (**top right**), and RNFL deviation map (**bottom right**) show sectoral RNFL measurements with color-coded normative comparison (green: within normal limits, yellow: borderline, red: outside normal limits); S—superior segment; T—temporal segment; N—nasal segment; I—inferior segment.

**Figure 2:**
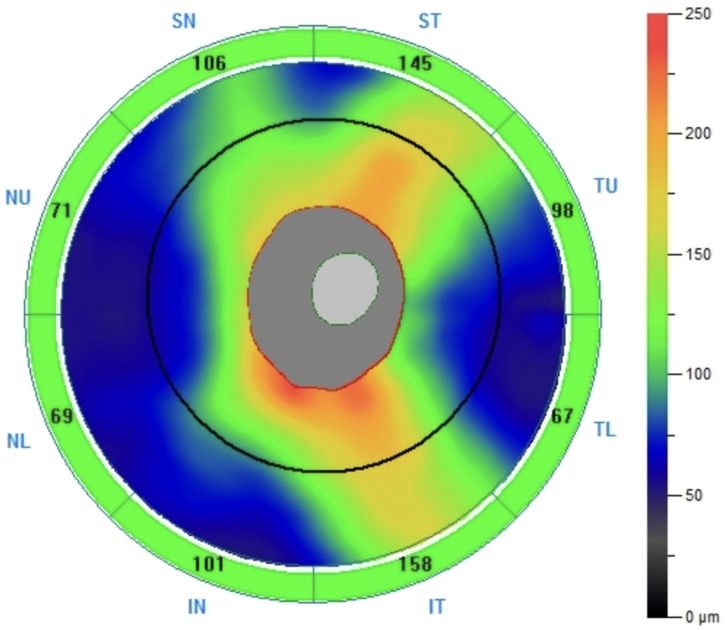
The map displays retinal nerve fiber layer thickness in individual sectors around the optic nerve head: ST—Superior Temporal; SN—Superior Nasal; NU—Nasal Upper; NL—Nasal Lower; IN—Inferior Nasal; IT—Inferior Temporal; TL—Temporal Lower; TU—Temporal Upper.

All patients in the study and control groups underwent optical coherence tomography (OCT) using the commercially available RTVue XR Avanti with AngioVue (Optovue, Fremont, CA, USA, www.optovue.com). The examinations included in the analysis were performed between 1.5 and 5 years after surgery, at a time when RNFL thickness had stabilized and was no longer showing signs of deterioration. The average interval between surgery and the OCT examination was approximately 3 years and 8 months. The imaging protocol employed in this study was the ONH (optic nerve head) protocol. All measurements were obtained using the automated default segmentation settings of the ONH protocol, which evaluates peripapillary thickness within a 4.0 mm circle diameter. Additionally, the protocol measures the mean peripapillary retinal nerve fiber layer (avgRNFL) thickness, as well as RNFL thickness in the upper segment (supRNFL), lower segment (infRNFL) (Figure 1), and in the four peripapillary sectors of the superior hemiretina Temporal Upper (TU), Superior Temporal (ST), Superior Nasal (SN), Nasal Upper (NU) and the inferior hemiretina Nasal Lower (NL), Inferior Nasal (IN), Inferior Temporal (IT), and Temporal Lower (TL) (Figure 2). The SupRNFL parameter is calculated as the average of the RNFL thickness measurements from the four sectors in the superior hemisphere, whereas the InfRNFL is the average from the four sectors in the inferior hemisphere. All acquired images were carefully analyzed. In cases where automatic segmentation errors or measurement artifacts were detected, the scan was rescanned. OCT scans affected by motion artifacts with a low signal quality index were excluded from the analysis.

All study group patients underwent comprehensive evaluations before and after surgery. The preoperative assessments included the following parameters: gender, place of birth, and age at the time of CP diagnosis. Tumor characteristics were also examined, including location (intrasellar and suprasellar/suprasellar/intrasellar), tumor volume, maximum tumor diameter, morphology (solid/cystic/mixed), calcifications, invading the third ventricle, cavernous sinus infiltration, hydrocephalus, and ventriculoperitoneal shunt. CPs are located either in the sella turcica (that is, the depression in the sphenoid bone, containing the pituitary gland; intrasellar) or above the sella turcica (suprasellar), or both intrasellar and suprasellar [1].

After surgery, we analyzed the surgical technique (bifrontal craniotomy, transcortical-transforaminal craniotomy), the degree of surgical resection (complete/incomplete), the presence of Rosenthal fibers, the need for reoperation due to recurrence or progression, the reason for reoperation, and the use of radiotherapy. Recurrence was defined as the reappearance of a tumor after complete resection, confirmed by postoperative magnetic resonance imaging or computed tomography. Progression was defined as an increase in the size of the residual tumor after partial resection, with or without clinical symptoms, requiring further treatment. Following surgery, all patients exhibited hypopituitarism, and hormone replacement therapy was assessed. The detailed characteristics of the study group are presented in Table 1.

### Statistical Analysis

The analysis was performed using Statistica software version 10 (StatSoft Inc., Tulsa, OK, USA). The Shapiro–Wilk test was used to assess the normality of the distribution of the analyzed variables. Categorical data are presented as counts and percentages, while continuous data are shown as medians with interquartile ranges (IQRs) and ranges, including minimum and maximum values. The Mann–Whitney test was applied to compare two groups, and ANOVA with Fisher’s least significant difference test was used for comparisons involving more than two groups. Spearman’s R-value was calculated for correlation analysis. A *p*-value of less than 0.05 was considered statistically significant.

## 3. Results

The data for 73 eyes of 38 patients with CP (mean age 10.3 ± 4.2 years, range 4–17; 22M/16F), and 64 eyes of 32 healthy, sex- and age-matched controls (mean age 10.5 ± 3.1 years; range 4–17; 12M/20F) were included in the analyses. The mean age at CP diagnosis was 8.5 ± 3.9 years (range: 1.9–16 years). The mean avgRNFL, supRNFL, infRNFL, and RNFL in all quadrants were statistically significantly thinner in the CP patient group compared to the control group.

Significant differences were observed in avgRNFL (79 μm, range 52–117 vs. 106 μm, range 93–137, *p* < 0.001), supRNFL (81 μm, range 50–120 vs. 107 μm, range 93–158, *p* < 0.001), and infRNFL (77 μm, range 49–120 vs. 102 μm, range 90–128, *p* < 0.001) between patients with CP after neurosurgical treatment and healthy children. A significant difference was also observed in almost all quadrants of the RNFL: ST (117 μm, range 55–162 vs. 141 μm, range 96–212, *p* < 0.001), TU (60 μm, range 36–101 vs. 89 μm, range 69–120, *p* < 0.001), TL (51 μm, range 29–89 vs. 89 μm, range 69–120, *p* < 0.001), IT (113 μm, range 53–172 vs. 147 μm, range 124–189, *p* < 0.001), IN (87 μm, range 11–156 vs. 114 μm, range 76–153, *p* < 0.001), NL (56 μm, range 31–114 vs. 77 μm, range 56–96, *p* < 0.001), NU (61 μm, range 29–126 vs. 88 μm, range 56–113, *p* < 0.001), and SN (85 μm, range 43–153 vs. 113 μm, range 72–200, *p* < 0.001). The difference in RNFL thickness per sector between CP children and the control group was as follows: ST 24 μm, TU 29 μm, TL 38 μm, IT 34 μm, IN 27 μm, NL 21 μm, NU 27 μm, and SN 28 μm, respectively. There was no correlation between the location of the affected segments and the degree of RNFL damage within those segments. The median, lower quartile, upper quartile, and ranges for avgRNFL, supRNFL, infRNFL, ST, TU, TL, IT, IN, NL, NU, and SN in the study group and the control group are presented in Table 2.

**Figure 3:**
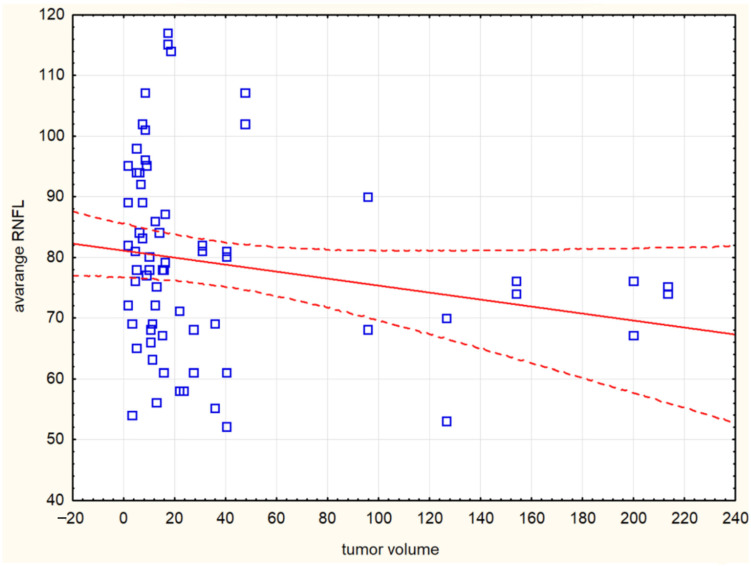
Correlation plot between tumor volume (cm^3^) and average retinal nerve fiber layer (AvgRNFL) thickness (*p* < 0.01; r = −0.3). Blue squares represent the individual data points. The solid red line indicates the linear regression fit, while the dashed red lines represent the 95% confidence interval for the regression estimate.

**Figure 4:**
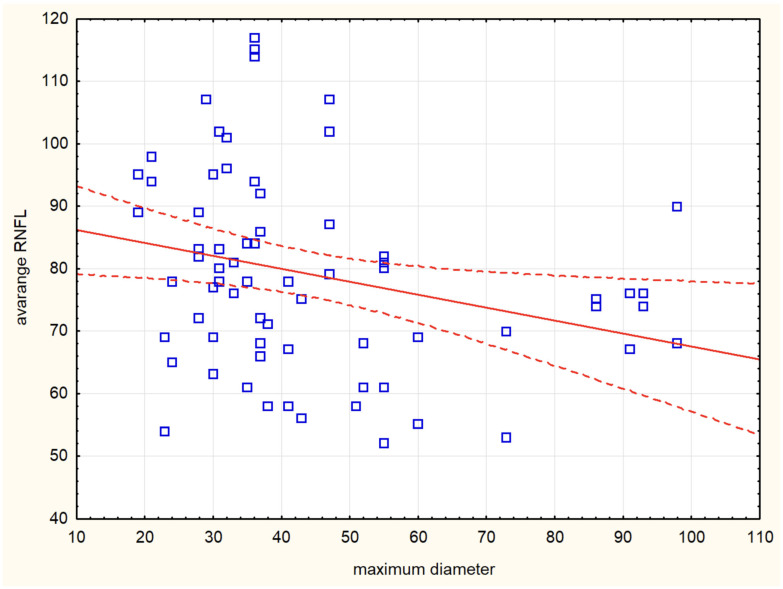
Correlation plot between maximum tumor diameter (mm) and average retinal nerve fiber layer (AvgRNFL) thickness (*p* < 0.01; r = −0.4). Blue squares represent the individual data points. The solid red line indicates the linear regression fit, while the dashed red lines represent the 95% confidence interval for the regression estimate.

The combined intrasellar and suprasellar tumor location had a statistically significant effect on the NL sector (*p* = 0.004). However, when comparing exclusively suprasellar versus exclusively intrasellar tumor locations, no significant differences were observed in any of the RNFL parameters. Tumor volume correlated with avgRNFL (r = −0.313) (Figure 3), supRNFL (r = −0.283), infRNFL (r = −0.34), IT (r = −0.358), IN (r = −0.376), NU (r = −0.252), SN (r = −0.324). Maximum tumor diameter correlated with avgRNFL (r = −0.345) (Figure 4), supRNFL (r = −0.299), infRNFL (r = −0.387), IT (r = −0.315), IN (r = 0.448), NL (r = −0.252), NU (r = −0.311), SN (r = −0.352). A larger maximum tumor diameter and tumor volume were associated with greater damage to the RNFL.

Calcification had a statistically significant effect on avgRNFL (*p* = 0.037), supRNFL (*p* = 0.027), infRNFL (*p* = 0.042), TU (*p* = 0.029), IN (*p* = 0.037), NL (*p* = 0.009), and NU (*p* = 0.015). The presence of calcification was associated with greater damage to RNFL fiber thickness in these sectors. The presence of a ventriculoperitoneal shunt impacted TU (*p* = 0.006) and TL (*p* = 0.005), resulting in more pronounced RNFL thinning in these regions.

In the group of CP patients who underwent bifrontal craniotomy, avgRNFL (76 μm vs. 104.5 μm, *p* = 0.003), supRNFL (78 μm vs. 108 μm *p* = 0.001), infRNFL (74 μm vs. 101 μm, *p* = 0.022), IN (79 μm vs. 133 μm, *p* = 0.002), NL (51 μm vs. 76 μm, *p* = 0.002) NU (55 μm vs. 89 μm *p* = 0.004), SN (81 μm vs. 118,5 μm, *p* = 0.002) were statistically significantly thinner than in the transcortical-transforaminal craniotomy group. No statistically significant differences were found for ST, TU, TL, and IT.

**Figure 5:**
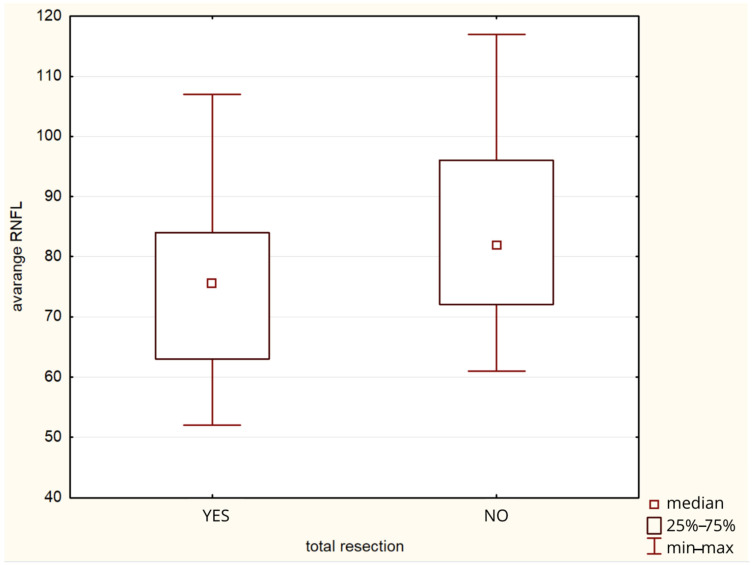
Graph of the influence of complete tumor resection on the average RNFL thickness.

Total tumor resection significantly influenced avgRNFL (*p* = 0.0026) (Figure 5), supRNFL (*p* = 0.031), infRNFL (*p* = 0.036), TU (*p* = 0.032), IT (*p* = 0.022), NL (*p* = 0.049), and SN (*p* = 0.024). Total tumor resection was associated with more extensive damage and a reduction in RNFL thickness. The presence of Rosenthal fibers affected NL (*p* = 0.036). Reoperation due to recurrence or progression of the CP affected TU (*p* = 0.027), IN (*p* = 0.023), NL (*p* = 0.045). Table 3.

Clinical parameters such as gender, place of birth, and age at CP diagnosis did not affect RNFL parameters. Tumor characteristics encompassing location, morphology (cystic, solid, mixed), invasion of the third ventricle, cavernous sinus infiltration, hydrocephalus and the reason for the reoperation also had no significant impact on RNFL measurements at individual sites. Radiotherapy did not affect RNFL parameters in individual sectors.

## 4. Discussion

Our study provides new evidence of a link between craniopharyngioma-induced compression of the optic chiasm and changes in RNFL thickness. This is the first study to assess multiple clinical parameters and tumor characteristics, as well as their impact on RNFL, in a pediatric patient group following neurosurgical procedures for CP. Optic nerve damage in patients with CP can be caused by direct compression, chronic swelling of the optic disc, surgical manipulation, or radiotherapy. The primary cause of neuronal cell damage, including RNFL thinning, in the case of sellar tumors, is considered to be direct compression of the optic chiasm. Retrograde degeneration has been identified as the underlying cause of retinal layer thinning observed in chiasmal compression and other optic neuropathies [27]. Increasing evidence suggests that this degeneration occurs in both retrograde and anterograde manners [27]. Damage to the RNFL is associated with structural and functional deterioration of the visual pathway, leading to transsynaptic degeneration [23,28]. Initially, compression leads to functional impairment, which is reflected as visual field damage. Retinal ganglion cell death occurs after prolonged compression, and there is a time delay before OCT detects RNFL thinning. In addition to direct compression, surgical treatment and complete resection of the tumor carry a high risk of vision loss due to direct damage to the visual structures or disruption of their vascularization [1,29,30,31]. In our study, we observed statistically significant differences in RNFL thinning across all analyzed locations of the optic nerve disc in CP patients compared to healthy children. This aligns with previous research findings that pressure on the optic nerve chiasm in patients with CP causes damage to optic nerve cells and the retina [7,11,25,32]. In contrast, no study has assessed the impact of tumor characteristics on RNFL damage in such a large group of children. Numerous studies have analyzed the correlation between RNFL thickness and functional visual parameters, such as visual acuity and visual field losses, in patients with optic chiasm compression [16,17,20,21,25,33,34,35,36,37]. Based on OCT, a correlation has been demonstrated between reduced RNFL thickness and visual field loss on perimetric examination in patients with optic chiasm compression [16,17,21,25,33,35,37], as well as a correlation with visual acuity [17,33,36]. Bialer et al. [25] demonstrated RNFL thinning in children with CP, with the degree of damage correlated with visual acuity and visual field loss. Similarly, Lee et al. [11] also evaluated the RNFL thickness after surgery in 18 pediatric patients with CP and reported thinning in all sectors. Mediero et al. [7] also reported a significant association between retinal ganglion cell complex, RNFL thickness, and visual field defects in 10 pediatric patients with CP. Yang et al. [26] reported RNFL changes in CP patients, noting that the damage was more pronounced in adult patients compared to pediatric patients. However, Qiao et al. [16] evaluated the relationship between OCT parameters and visual parameters in 88 adult patients before tumor removal by endoscopic endonasal surgery. They observed that a greater thickness in the temporal segment is a positive factor for a favorable prognosis of visual acuity. In contrast, a thicker thickness in the inferior sector is an independent prognostic indicator for visual field loss.

Given that optic nerve fibers cross at the optic chiasm, compression from a craniopharyngioma occurs at this location. It is logical to expect more significant damage in the nasal regions compared to the temporal ones. A characteristic outcome of pressure at the optic chiasm is temporal hemianopia. Akashi et al. [24], based on their analysis, reached this conclusion, observing more pronounced damage to the macular RNFL (mRNFL) and ganglion cell layer in the nasal hemiretina. This specific pattern of nasal damage may help distinguish chiasmal compression from other conditions with distinct patterns of ganglion cell loss, such as glaucoma. Compression of the optic chiasm can also lead to RNFL thinning in the nasal and temporal sectors of the peripapillary retina, while relatively preserving the superior and inferior sectors [22,38]. This type of RNFL degeneration is referred to as band atrophy of the optic nerve. It contrasts with the predominant thinning of the superior and inferior regions seen in glaucoma-affected eyes [22,38]. However, Lee et al. [11] analyzed the RNFL in 18 patients with juvenile CP after surgery. They noted statistically significant thinning in almost all quadrants, namely the superior, inferior, and nasal quadrants. However, they did not observe a statistically significant difference in RNFL thinning in the temporal segment. In our study, however, no significant difference in damage was observed between the nasal and temporal sectors of the RNFL. This may be attributed to the extensive damage and advanced stage of the lesions in pediatric patients, which could obscure any regional differences. Another potential explanation is that our evaluation was conducted post-surgery rather than before it, which may have masked any initial patterns of nasal sector damage. However, tumor size, as expressed by tumor volume, showed a significant correlation with RNFL thinning in nasal optic nerve sectors: IT, IN, NU, and SN. Similarly, maximum tumor diameter correlated with thinning in the nasal sectors: IT, IN, NU, SN, and NL. This is consistent with the above theory and the fact that nasal fibers, located closer to and more centrally within the site of optic nerve compression, are the first to be affected [21,22,39].

This study found that children who underwent bifrontal surgery for CP had worse outcomes in RNFL thickness compared to those who underwent a transcortical-transforaminal approach. The transcortical-transforaminal route is typically used for CP located within the third ventricle, which are tumors that are very rare and anatomically more distant from the optic chiasm. The lesser RNFL damage observed in these cases may be attributed primarily to this greater distance from the optic pathways. In contrast, the bifrontal approach is typically used in cases where the tumor is located in the intra- and suprasellar regions, placing it close to the optic chiasm. As a result, these tumors are more likely to cause direct damage to the RNFL through mechanical compression [40,41].

Orski et al. [40] studied the effect of robotic fractionated radiation therapy on tumors of the parasellar region, including CP, on the structure and function of the eye. They noted the impact of the radiotherapy on RNFL cells and their thinning. In contrast, in line with the systematic review, Colliander et al. [6] reported that patients with CP after radiotherapy most often had impaired visual function but no RNFL. Conversely, in our study, radiotherapy did not affect the severity of RFNL damage in patients after CP surgery. This discovery is particularly noteworthy given that radiotherapy has the potential to damage the visual pathways, which can lead to complications such as radiation-induced optic neuropathy, retinopathy, optic pathway necrosis, and ocular toxicity [41,42]. Other damaging factors, directly related to the presence of the tumor earlier in the course of the disease and treatment, may have contributed to the absence of observed effects of radiotherapy on the RNFL.

Several limitations of this study should be acknowledged. First, the retrospective nature of the study may introduce selection bias. Second, the sample size, while adequate for preliminary analysis, may limit the generalizability of the findings. Additionally, the study did not account for potential confounding factors, such as variations in surgical techniques or postoperative care, which could influence RNFL outcomes. Furthermore, poorly cooperative patients, most often the youngest children, and those who were unable to maintain stable fixation due to significantly reduced visual acuity, were excluded from the study. As a result, OCT could not be performed in the most severely affected cases, introducing a potential selection bias by excluding eyes with the greatest degree of structural damage.

Our results suggest that all patients, without exception, exhibit RNFL thinning in all peripapillary sectors following neurosurgery for craniopharyngioma. This widespread thinning may indicate irreversible axonal damage and highlights the importance of long-term visual monitoring in this patient population. This study reinforces the value of OCT as a non-invasive, objective tool for assessing optic nerve integrity in pediatric CP patients. Quantifying RNFL thickness allows clinicians to detect subclinical lesions very early, which is particularly valuable in pediatric patients. OCT could benefit long-term follow-up and monitoring to identify patients at risk of progressive visual deterioration and guide targeted interventions.

## 5. Conclusions

This study demonstrates that pediatric patients with CP experience significant thinning of the peripapillary RNFL following neurosurgery. The OCT enables objective long-term monitoring, offering advantages over subjective visual acuity assessments in children. Further research is needed to investigate the influence of individual tumor characteristics and clinical factors on the degree of RNFL damage.

This manuscript was developed with support from the Medical Research Agency (ABM) as part of an educational program. It is a product of the Polish Clinical Scholar Training, organized in collaboration with Harvard Medical School’s Postgraduate Medical Education. The content of this manuscript represents the author’s work and is not affiliated with or endorsed by Harvard Medical School.

## Figures and Tables

**Table 1:** Characteristics of the study group population of 73 eyes of 38 patients, divided into patients and eyes diagnosed with childhood-onset, adamantinomatous craniopharyngioma (CP) and recruited in the study. Categorical variables are presented as count *n* and the corresponding percentage (%).

Craniopharyngioma (CP) Patient Characteristics	Study Cohort (Patients)	Study Cohort (Eyes)
	*n* (%)	*n* (%)
Sex, female/male, %	16 (42%)/22 (58%)	31 (42%)/42 (58%)
Place of birth, city/country	28 (74%)/10 (26%)	53 (73%)/20 (27%)
Mean age at CP diagnosis, years (range)	8.5 (1.9–16)	
Mean age at OCT examination, years (range)	10.3 (4–17)	
Tumor location		
Intrasellar and suprasellar	24 (63%)	46 (63%)
Suprasellar	14 (37%)	27 (37%)
Intrasellar	0 (0%)	0 (0%)
Median tumor volume, cm^3^ (range)	35.2 (1.8–213.7)	
Median maximum tumor diameter, mm (range)	44.2 (19–98)	
Tumor morphology solid/cystic	4 (11%)/34 (89%)	7 (10%)/66 (90%)
Calcifications	37 (97%)	71 (97%)
Invading the third ventricle	29 (76%)	55 (78%)
Cavernous sinus infiltration	2 (5%)	4 (5%)
Hydrocephalus	16 (42%)	29 (40%)
Ventriculoperitoneal shunt	6 (16%)	12 (16%)
Bifrontal craniotomy/transcortical-transforaminal craniotomy	31 (87%)/5 (13%)	63 (86%)/10 (14%)
Degree of surgical resection (complete/incomplete resection)	23 (61%)/15 (39%)	46 (63%)/27 (37%)
Rosenthal fibers in histopathology examination	5 (13%)	9 (12%)
Progression	5 (13)	9 (12%)
Recurrence	4 (11%)	7 (10%)
Reoperation	7 (8%)	13 (18%)
Radiotherapy	10 (24%)	19 (26%)

**Table 2:** Descriptive statistics for retinal nerve fiber layer RNFL (μm) at selected anatomical locations (patients with CP vs. controls). M: median; Q1 lower quartile represents the 25th percentile of the data; Q3 upper quartile corresponds to the 75th percentile; *p*-value: statistical significance value; avgRNFL: average retinal nerve fiber layer; supGCC: superior retinal nerve fiber layer; infGCC: inferior retinal nerve fiber layer; TU: temporal upper retinal nerve fiber layer, ST: superior temporal retinal nerve fiber layer, SN: superior nasal retinal nerve fiber layer, NU: nasal upper retinal nerve fiber layer, NL: nasal lower retinal nerve fiber layer, IN: inferior nasal retinal nerve fiber layer, IT: inferior temporal retinal nerve fiber layer, TL: temporal lower retinal nerve fiber layer. The Mann–Whitney test was used to compare RNFL parameters in the control and study groups.

Variable	Craniopharyngioma Group				Control Group				*p*
	M	Q1	Q3	Range	M	Q1	Q3	Range	
avgRNFL (μm)	78	68	89	52–117	104	101	109	93–137	<0.001
supRNFL (μm)	78	69	91	50–120	104	102	111.5	93–158	<0.001
infRNFL (μm)	78	63	86	49–120	102	98	108.5	90–128	<0.001
ST (μm)	117	103	131	55–162	139.5	130	150.5	55–162	<0.001
TU (μm)	59	48	70	36–101	87.5	81	95.5	69–120	<0.001
TL (μm)	51	38	59	29–91	74	69.5	79.5	57–105	<0.001
IT (μm)	113	99	130	53–172	145	137.5	153.5	124–189	<0.001
IN (μm)	80	64	106	111–156	112.5	104	123	76–153	<0.001
NL (μm)	53	44	65	31–114	77	71.5	83	56–96	<0.001
NU (μm)	57	47	68	29–126	88	81.5	95	56–113	<0.001
SN (μm)	82	67	95	43–153	110.5	100	124	72–200	<0.001

**Table 3:** Impact of selected clinical and surgical factors on RNFL thickness in pediatric patients with craniopharyngioma. Statistically significant differences are presented for each RNFL sector (avgRNFL—average retinal nerve fiber layer; supRNFL—superior RNFL; infRNFL—inferior RNFL; TU—temporal upper sector; TL—temporal lower sector; IN—inferior nasal sector; IT—inferior temporal sector; SN—superior nasal sector; ST—superior temporal sector; NL—nasal lower sector; NU—nasal upper sector) depending on the presence of calcification, ventriculoperitoneal shunt, type of craniotomy, extent of tumor resection, presence of Rosenthal fibers, and reoperation due to tumor recurrence. Values represent sectors with significant RNFL thinning (*p* < 0.05).

Clinical Factor	RNFL Parameter	Effect	*p*-Value
Calcification	avgRNFL	Thinner with calcification	0.037
	supRNFL	Thinner with calcification	0.027
	infRNFL	Thinner with calcification	0.042
	TU	Thinner with calcification	0.029
	IN	Thinner with calcification	0.037
	NL	Thinner with calcification	0.009
	NU	Thinner with calcification	0.015
Ventriculoperitoneal shunt	TU	Thinner in shunted patients	0.006
	TL	Thinner in shunted patients	0.005
Craniotomy type	avgRNFL	Bifrontal < Transcortical	0.003
	supRNFL	Bifrontal < Transcortical	0.001
	infRNFL	Bifrontal < Transcortical	0.022
	IN	Bifrontal < Transcortical	0.002
	NL	Bifrontal < Transcortical	0.002
	NU	Bifrontal < Transcortical	0.004
	SN	Bifrontal < Transcortical	0.002
	ST, TU, TL, IT	No significant difference	n.s.
Total tumor resection	avgRNFL	Thinner after total resection	0.0026
	supRNFL	Thinner after total resection	0.031
	infRNFL	Thinner after total resection	0.036
	TU	Thinner after total resection	0.032
	IT	Thinner after total resection	0.022
	NL	Thinner after total resection	0.049
	SN	Thinner after total resection	0.024
Rosenthal fibers	NL	Thinner when Rosenthal fibers present	0.036
Reoperation	TU	Thinner in reoperated patients	0.027
	IN	Thinner in reoperated patients	0.023
	NL	Thinner in reoperated patients	0.045

## Data Availability

The data presented in this study are available on request from the corresponding author. The data are not publicly available due to ethical restrictions and the need to protect patient confidentiality.
